# Electronic nose homogeneous data sets for beef quality classification and microbial population prediction

**DOI:** 10.1186/s13104-022-06126-9

**Published:** 2022-07-07

**Authors:** Dedy Rahman Wijaya, Riyanarto Sarno, Enny Zulaika, Farah Afianti

**Affiliations:** 1 School of Applied Science, Telkom University, Jalan Telekomunikasi Terusan Buah Batu, Bandung, West Java Indonesia; 2 Department of Informatics, Faculty of Intelligent Electrical and Informatics Technology, Institut Teknologi Sepuluh Nopember (ITS) Sukolilo, Surabaya, Indonesia; 3 Department of Biology, Institut Teknologi Sepuluh Nopember, Jalan Raya ITS, Keputih, Sukolilo, 60111 Surabaya, East Java Indonesia; 4 School of Computing, Telkom University, Jalan Telekomunikasi Terusan Buah Batu, Bandung, West Java Indonesia

**Keywords:** Electronic nose, Gas sensor, Homogeneous data sets, Beef quality, Machine learning

## Abstract

**Objectives:**

In recent years, research on the use of electronic noses (e-nose) has developed rapidly, especially in the medical and food fields. Typically, e-nose is coupled with machine learning algorithms to detect or predict multiple sensory classes in a given sample. In many cases, comprehensive and complete experiments are required to ensure the generalizability of the predictive model. For this reason, homogeneous data sets are important to use. Homogeneous data sets refer to the data sets obtained from different observations in almost similar environmental condition. In this data article, e-nose homogeneous data sets are provided for beef quality classification and microbial population prediction.

**Data description:**

This data set is originated from 12 type of beef cuts. The process of beef spoilage is recorded using 11 Metal-Oxide Semiconductor (MOS) gas sensors for 2220 min. The formal standards, issued by the Meat Standards Committee, are used as a reference in labeling beef quality. Based on the number of microbial populations, meat quality was grouped into four classes, namely excellent, good, acceptable, and spoiled. The data set is formatted in "xlsx" file. Each sheet represents one beef cut. Moreover, data sets are good cases for feature selection algorithm stability test, especially to solve sensor array optimization problems.

## Objective

In recent years, research on the use of the e-nose has developed, especially in the medical and food fields. Typically, e-noses are combined with machine learning algorithm to detect or predict several sensory classes in a particular sample. Hence, e-nose signal processing becomes an essential part of e-nose systems. In many cases, comprehensive and complete experiments are required to make sure the generalization of prediction model. Even though they have almost the same pattern, the combination of several experiments shows that there are differences in the homogeneous data sets. Based on several machine learning algorithms, experimental results show different scores with different cuts of beef [1]. This difference can be influenced by the composition or mixture of samples (protein, lipid, etc.) and also environmental factors, for example temperature and humidity. Using a comprehensive data set is one of the ways needed to build a predictive model that has good generalization. In this data article, homogeneous data sets refer to the data sets collected from different samples in almost similar environmental conditions. The homogeneous data sets are suitable for developing and testing the generalizability of machine learning models [2]. The availability of homogeneous data sets will provide a more comprehensive pattern, especially regarding the assessment of beef quality with various types of beef cuts compared to other data sets [3]. Moreover, these data sets are good cases for feature selection algorithm (FSA) stability tests [4], especially to overcome sensor array optimization problems in e-nose [5].

## Data description

This data set is originated from 12 type of beef cuts such as tenderloin, striploin (shortloin), top sirloin, brisket, rib eye, skirt meat (plate), round (shank), inside/outside, chuck/ clod, fat, shin, and flank (flap meat). The process of beef spoilage is recorded using 11 gas sensors of Metal-Oxide Semiconductor (MOS) for 2220 min. The data set is formatted in "xlsx" file. Each sheet represents one beef cut which is a contained column as follows [6]:

- Minute: time in minute
- TVC: continuous label in the total viable count (microbial population)
- Label: discrete label, 1,2,3,4 denote “excellent”, ”good”, ”acceptable”, and “spoiled”, respectively.
- MQ_: the resistant value of gas sensors.

Table 1 shows the overview of data sets. Formal standards, issued by the Meat Standards Committee of Agricultural and Resource Management Council of Australia and New Zealand (ARMCANZ), are used for labeling beef quality [7]. The optical density (600 nm) is measured by a Spectrophotometer (Genesys 20), and the number of cells is calculated by a hemocytometer. Classical and two-hour methods were applied in the experiment [8]. Based on the number of microbial populations (log_10_ cfu/g), meat quality was grouped into four classes, namely excellent, good, acceptable, and spoiled.

**Table 1:** Brief description of data set

Label	Name of data set	File types (file extension)	Data repository and identifier (DOI or accession number)
Data file 1	e-nose_data set_12_beef_cuts	MS Excel file (.xlsx)	Harvard Dataverse (https://doi.org/10.7910/DVN/XNFVTS) [6]
Data file 2	Component list for experiment	MS Excel file (.xlsx)	Harvard Dataverse (https://doi.org/10.7910/DVN/XNFVTS) [6]
Data file 3	Experiment	Image (.jpg)	Harvard Dataverse (https://doi.org/10.7910/DVN/XNFVTS) [6]
Data file 4	Brisket	Image (.jpg)	Harvard Dataverse (https://doi.org/10.7910/DVN/XNFVTS) [6]
Data file 5	clod-chuck	Image (.jpg)	Harvard Dataverse (https://doi.org/10.7910/DVN/XNFVTS) [6]
Data file 6	Fat	Image (.jpg)	Harvard Dataverse (https://doi.org/10.7910/DVN/XNFVTS) [6]
Data file 7	flap meat	Image (.jpg)	Harvard Dataverse (https://doi.org/10.7910/DVN/XNFVTS) [6]
Data file 8	inside-outside	Image (.jpg)	Harvard Dataverse (https://doi.org/10.7910/DVN/XNFVTS) [6]
Data file 9	rib eye	Image (.jpg)	Harvard Dataverse (https://doi.org/10.7910/DVN/XNFVTS) [6]
Data file 10	Round	Image (.jpg)	Harvard Dataverse (https://doi.org/10.7910/DVN/XNFVTS) [6]
Data file 11	Shin	Image (.jpg)	Harvard Dataverse (https://doi.org/10.7910/DVN/XNFVTS) [6]
Data file 12	skirt meat	Image (.jpg)	Harvard Dataverse (https://doi.org/10.7910/DVN/XNFVTS) [6]
Data file 13	Striploin	Image (.jpg)	Harvard Dataverse (https://doi.org/10.7910/DVN/XNFVTS) [6]
Data file 14	Tenderloin	Image (.jpg)	Harvard Dataverse (https://doi.org/10.7910/DVN/XNFVTS) [6]
Data file 15	top sirloin	Image (.jpg)	Harvard Dataverse (https://doi.org/10.7910/DVN/XNFVTS) [6]

The data sets were generated from the experimental results from the decomposition of several types of beef cuts recorded using a prototype mobile e-nose device at a stable temperature (Data file 1). A list of components used in the experiment can be seen in “Component list for experiment.xlsx”. The experiments are carried out with fresh meat from a shop or market with the assumption that the meat has the best level of freshness (excellent). The experiment was carried out at a controlled temperature (± 29 °C) with the aim of making the response of the gas sensor more stable. The experimental scheme at controlled temperature can be seen in Data file 3 [6]. The data generated by the gas sensor array in the sample chamber will be sent to the server automatically every minute to see the condition of the meat for 2–3 days non-stop. The weight of the meat tested was 125 g which were placed in the sample chamber for approximately 2 days until it reached a spoiled condition. In the experiment, the type of meat will also be considered based on the fat composition in the cut of meat. This data set is originated from 12 beef cuts types such as tenderloin, striploin (shortloin), top sirloin, brisket, rib eye, skirt meat (plate), round (shank), inside/outside, chuck/ clod, fat, shin, and flank (flap meat) as shown in Data file 4 to Data file 15 [6].

## Limitations

These data sets are collected at controlled temperatures. However, humidity is uncontrolled and naturally affected by the air vapor from the meat decomposition process which may affect noise contamination in the data sets.

## Data Availability

The data described in this Data note can be freely and openly accessible on *Harvard Dataverse *under https://doi.org/10.7910/DVN/XNFVTS. Please see Table 1 and references [6] for details and links to the data.

## Abbreviations

e-nose: electronic nose
MOS: metal-oxide semiconductor
TVC: total viable count
FSA: feature selection algorithm
